# Stigma and Web-Based Sex Seeking Among Men Who Have Sex With Men and Transgender Women in Tijuana, Mexico: Cross-Sectional Study

**DOI:** 10.2196/14803

**Published:** 2020-01-30

**Authors:** Cristina Espinosa da Silva, Laramie R Smith, Thomas L Patterson, Shirley J Semple, Alicia Harvey-Vera, Stephanie Nunes, Gudelia Rangel, Heather A Pines

**Affiliations:** 1 Department of Medicine University of California, San Diego La Jolla, CA United States; 2 Department of Psychiatry University of California, San Diego La Jolla, CA United States; 3 Universidad Xochicalco Tijuana Mexico; 4 United States-Mexico Border Health Commission Tijuana Mexico; 5 El Colegio de la Frontera Norte Tijuana Mexico

**Keywords:** social stigma, discrimination, internet, mobile apps, men who have sex with men, transgender persons, sexual and gender minorities, Mexico

## Abstract

**Background:**

Stigma toward sexual and gender minorities is an important structural driver of HIV epidemics among men who have sex with men (MSM) and transgender women (TW) globally. Sex-seeking websites and apps are popular among MSM and TW. Interventions delivered via Web-based sex-seeking platforms may be particularly effective for engaging MSM and TW in HIV prevention and treatment services in settings with widespread stigma toward these vulnerable populations.

**Objective:**

To assess the potential utility of this approach, the objectives of our study were to determine the prevalence of Web-based sex seeking and examine the effect of factors that shape or are influenced by stigma toward sexual and gender minorities on Web-based sex seeking among MSM and TW in Tijuana, Mexico.

**Methods:**

From 2015 to 2018, 529 MSM and 32 TW were recruited through venue-based and respondent-driven sampling. Interviewer-administered surveys collected information on Web-based sex seeking (past 4 months) and factors that shape or are influenced by stigma toward sexual and gender minorities (among MSM and TW: traditional machismo, internalized stigma related to same-sex sexual behavior or gender identity, and outness related to same-sex sexual behavior or gender identity; among MSM only: sexual orientation and history of discrimination related to same-sex sexual behavior). A total of 5 separate multivariable logistic regression models were used to examine the effect of each stigma measure on Web-based sex seeking.

**Results:**

A total of 29.4% (165/561) of our sample reported seeking sex partners on the Web. Web-based sex seeking was negatively associated with greater endorsement of traditional machismo values (adjusted odds ratio [AOR] 0.36, 95% CI 0.19 to 0.69) and greater levels of internalized stigma (AOR 0.96, 95% CI 0.94 to 0.99). Web-based sex seeking was positively associated with identifying as gay (AOR 2.13, 95% CI 1.36 to 3.33), greater outness (AOR 1.17, 95% CI 1.06 to 1.28), and a history of discrimination (AOR 1.83, 95% CI 1.08 to 3.08).

**Conclusions:**

Web-based sex-seeking is relatively common among MSM and TW in Tijuana, suggesting that it may be feasible to leverage Web-based sex-seeking platforms to engage these vulnerable populations in HIV prevention and treatment services. However, HIV interventions delivered through Web-based sex-seeking platforms may have limited reach among those most affected by stigma toward sexual and gender minorities (ie, those who express greater endorsement of traditional machismo values, greater levels of internalized stigma, lesser outness, and nongay identification), given that within our sample they were least likely to seek sex on the Web.

## Introduction

### Background

Men who have sex with men (MSM) and transgender women (TW) are at increased risk of HIV infection worldwide, including those in low- and middle-income countries (LMIC) [1,2]. Stigma toward sexual and gender minorities is an important structural driver of the HIV epidemics among MSM and TW in LMIC, with evidence suggesting that such stigma is linked to increased sexual risk behaviors [3-6] and limits the provision and uptake of HIV prevention and treatment services within these socially marginalized populations [7-10]. Use of sex-seeking websites and mobile apps among MSM and TW has grown in popularity globally, with prevalence estimates ranging from 36% in Latin America [11], 44% in North America [12], 39% to 62% in Africa [13,14], and 41% to 77% in Asia [15-18]. This has sparked interest in and led to the development of interventions that harness these Web-based platforms to engage MSM and TW in safer sex practices [19], HIV testing [20,21], and HIV care [22]. Given that sex-seeking websites and apps allow MSM and TW to meet new sexual partners with more anonymity than physical venues where they may risk being outed, discriminated against, or harmed because of their sexual or gender identity [13,14], interventions delivered through Web-based sex-seeking platforms may be particularly effective for engaging MSM and TW in HIV prevention and treatment services in regions with widespread stigma toward these vulnerable populations [19-21].

Stigma stems from power structures that promote or maintain social inequity [23-25] and manifests at the structural, interpersonal, and individual levels [24,25] as prejudice and discrimination toward persons with socially devalued characteristics, such as sexual and gender minorities [23,24,26-28]. Sexual and gender minorities often face significant stigma in heteronormative societies where cultural values enforce traditional gender roles [23,26-29]. In these settings, sexual and gender minorities may experience discrimination [25,30], internalize or adopt negative feelings and shame about their own sexuality or gender identity over time [28], and anticipate future stigmatizing experiences [28,31], which may cause some to conceal their sexual or gender identity [32]. Although several exploratory studies in LMIC have examined the relationship between factors that shape or are influenced by stigma and Web-based sex seeking [11,13-18], findings are mixed regarding whether these Web-based platforms are being used by MSM who are more or less affected by stigma. Studies in Peru [11] and China [15,17,18] found that Web-based sex seekers were more likely to be MSM who identified as gay, which is more common among MSM less affected by stigma. Studies that measured a history of discrimination and perceived or anticipated stigma among MSM in Nigeria [14] and Vietnam [16], on the other hand, found that Web-based sex seeking was associated with a history of discrimination in Nigeria and perceived or anticipated stigma in both Nigeria and Vietnam, suggesting that Web-based sex seeking may be more common among MSM more affected by stigma. Furthermore, a study conducted among MSM in Swaziland and Lesotho [13] measured gay identification, history of discrimination, and perceived or anticipated stigma and found that all 3 were associated with Web-based sex seeking. However, few studies have examined how a range of factors that shape or are influenced by stigma are related to Web-based sex seeking, and less is known about the role of internalized stigma or social norms that perpetuate stigma toward sexual and gender minorities.

Mexico’s HIV epidemic is concentrated within key populations [33,34], including MSM and TW in the Mexico-United States border region. HIV prevalence among MSM and TW in Tijuana, which lies along Mexico’s northern border with San Diego, California, is estimated to be approximately 20%, with nearly 90% of those testing HIV positive reporting no prior knowledge of their HIV status [35,36]. Stigma toward sexual and gender minorities is common in Mexico [37-40] and may be partially attributed to the cultural norm of *machismo* [41-44], which defines rigid gender roles and reflects heteronormative expectations of male behavior [41,45]. Findings from a recent cross-sectional study in Tijuana suggest that MSM who are less out about their same-sex sexual behavior and have higher levels of internalized stigma are less likely to seek HIV testing, which may contribute to the high prevalence of undiagnosed HIV infection among sexual and gender minorities in this setting [46]. Given that MSM and TW most affected by stigma in Tijuana are least likely to undergo HIV testing, Web-based strategies may help increase their frequency of HIV testing and support their timely linkage to HIV prevention and treatment services. Web-based strategies may also represent a feasible method to increase uptake of these services, considering that 54% of adults in Mexico reported owning a smartphone or using the internet as of 2015 [47].

### Objectives

To assess the potential utility of this approach and inform the development of HIV prevention efforts for MSM and TW in Tijuana and other comparable low-income settings, we aimed to determine the prevalence of Web-based sex seeking and examine the effect of stigma on Web-based sex seeking among MSM and TW in Tijuana. We hypothesized that Web-based sex seeking would be more common among MSM and TW most affected by stigma toward sexual and gender minorities (ie, MSM and TW with greater endorsement of traditional *machismo* values, greater levels of internalized stigma related to same-sex sexual behavior or gender identity, and lesser outness about same-sex sexual behavior or gender identity and MSM who do not identify as gay or report a history of discrimination related to their same-sex sexual behavior).

## Methods

### Study Population and Design

Data for this analysis came from 2 studies conducted in Tijuana, *Proyecto Enlaces* (Links Project) and *Proyecto Redes* (Networks Project). *Proyecto Enlaces* was designed to compare the effectiveness of 2 recruitment methods—venue-based sampling (VBS) and respondent-driven sampling (RDS)—for the identification of undiagnosed HIV infection among MSM and TW. *Proyecto Redes* was embedded in *Proyecto Enlaces* and was designed to characterize the sexual networks of MSM and TW. *Proyecto Enlaces* was conducted between March 2015 and November 2018, whereas *Proyecto Redes* was conducted between March 2016 and September 2017.

VBS was performed using time-location sampling across 36 venues identified during formative research as locations frequented by MSM and TW in Tijuana (eg, nightclubs, bars, public spaces, and motels). Individuals identified via VBS were eligible for HIV testing if they were aged at least 18 years, cisgender male or transgender female, reported anal sex with a cisgender male or transgender female in the past 4 months, and did not report a previous HIV diagnosis. RDS is a chain-referral sampling technique often used to recruit hard-to-reach populations [48,49]. A total of 33 individuals identified through VBS or referrals from Tijuana’s municipal HIV clinic were selected to initiate RDS recruitment chains (ie, seeds). Seeds were further selected to be diverse with respect to HIV status, age, socioeconomic status, sexual orientation, gender identity, and recruitment venue. Individuals were eligible to be seeds if they were aged at least 18 years, cisgender male or transgender female, reported anal sex with a cisgender male or transgender female in the past 4 months, lived in Tijuana, and reported social networks that included at least 15 MSM or TW who also lived in Tijuana (changed to 5 MSM or TW in April 2017 to boost RDS recruitment). Seeds were given 3 coupons to recruit their MSM or TW peers (eg, sex partners, acquaintances, friends, and family members) to participate in the study. Peer recruits were then given 3 coupons to recruit peers themselves if they provided their referral coupon, were aged at least 18 years, were cisgender male or transgender female, and reported anal sex with a cisgender male or transgender female in the past 12 months. Those who did not report a previous HIV diagnosis were also eligible for HIV testing. Beginning in January 2018, seeds and peer recruits were given 6 coupons to boost RDS recruitment. A Microsoft Access database was used to track peer recruitment and store biometric information to prevent duplicate enrollment. Seeds and peer recruits were given Mexican pesos (Mxn) $100 (approximately US $5) for every eligible peer they referred to the study.

As individuals could be identified multiple times via the same or a different recruitment method, those who were identified more than once and who remained eligible for HIV testing were retested if it had been at least 3 months since their last test. Eligible individuals identified via VBS underwent rapid HIV testing (Advanced Quality HIV 1/2 Test Kits, Intec Products Inc) at recruitment venues or at the study site if they preferred, whereas those identified via RDS underwent rapid HIV testing at the study site. All rapid HIV test results and appropriate posttest counseling were delivered within a few days (VBS) or 20 min (RDS) at the study site. All rapid test–negative individuals identified via VBS and rapid test–negative individuals identified via RDS who reported anal sex with a cisgender male or transgender female in the past 4 months (for comparability with those identified via VBS) were offered enrollment in *Proyecto Redes*. Rapid test–positive individuals provided an additional blood sample for confirmatory testing via immunofluorescence assay at the San Diego County Public Health Laboratory and were offered enrollment in *Proyecto Enlaces*. Confirmatory HIV test results were delivered to rapid test–positive individuals within 2 weeks, and those confirmed HIV positive were referred for free HIV care at Tijuana’s municipal HIV clinic.

All study procedures occurred at the study site located in an unmarked office building staffed by local Spanish-speaking individuals with extensive experience working with sexual and gender minorities in Tijuana who were also trained to foster a nonjudgmental environment. In our previous research with key populations in Tijuana, our study site was recognized as a nonstigmatizing setting where participants felt comfortable visiting without fear of discrimination. All participants provided written informed consent, and all study procedures were approved by the Human Subjects Protection Committees at the Universidad de Xochicalco in Tijuana and the University of California, San Diego.

### Data Collection

Surveys collected sociodemographic, psychosocial, and behavioral data and were interviewer administered using computer-assisted personal interviewing.

#### Exposures of Interest

A total of 5 variables measured different factors that shape or are influenced by stigma toward sexual and gender minorities, including traditional *machismo,* internalized stigma related to same-sex sexual behavior or gender identity, sexual orientation, outness about same-sex sexual behavior or gender identity, and history of discrimination related to same-sex sexual behavior (only measured among MSM participants; Multimedia Appendix 1). *Machismo* is a common social norm in Mexico and is composed of positive and negative constructs (termed *caballerismo* and traditional *machismo*, respectively) [50]. Traditional *machismo* is relevant to this study because it reflects a heteronormative version of masculinities that may be at odds with stereotypes associated with sexual behaviors (eg, receptive anal sex) and the sexual or gender identity of MSM and TW, and it likely shapes stigma toward sexual and gender minorities in Mexico. Traditional *machismo* was measured using a 10-item scale [50,51] that captures the negative characteristics typically associated with *machismo,* including hypermasculinity, aggressivity, and being domineering (eg, “It is necessary for a man to fight when challenged” and “A man should be in control of his wife”; Cronbach alpha=.90). Participants indicated their level of agreement with these items using 4-point Likert scale responses (1=strongly disagree, 2=disagree, 3=agree, and 4=strongly agree). A mean score was calculated from item responses, with higher scores indicating greater endorsement of traditional *machismo* values [51]. Internalized stigma related to same-sex sexual behavior or gender identity refers to an individual’s internalization of society’s negative view of sexual and gender minorities. MSM participants were asked to provide their level of agreement with 9 items assessing internalized stigma toward same-sex sexual behaviors (eg, “I have tried to stop being attracted to men in general” and “I would like to get professional help in order to change my sexual attraction from men to women exclusively”) using 5-point Likert scale responses (1=strongly disagree, 2=somewhat disagree, 3=neither agree nor disagree, 4=agree, and 5=strongly agree; Cronbach alpha=.93) [52]. To measure internalized stigma related to gender identity, we adapted the 9 items presented to MSM to reflect experiences of TW (eg, “I have tried to stop being attracted to men in general” was changed to “I have tried to stop identifying as a woman in general”; Cronbach alpha=.89). Participants’ responses to items measuring internalized stigma were summed to create a score, where higher scores indicate greater levels of internalized stigma. Sexual orientation was assessed by asking participants, “What is your sexual orientation?” (1=gay or homosexual, 2=heterosexual, 3=bisexual, and 4=not sure). Participants who reported being gay or homosexual were classified as gay identifying and all others were considered nongay identifying. We hypothesized that participants may have reported being unsure of their sexual orientation because of greater levels of internalized stigma. Owing to the small number of participants who reported being unsure of their sexual orientation (only 13 MSM participants), we combined this group with other MSM hypothesized to be more affected by stigma (ie, nongay identifying). Outness was measured by asking MSM and TW to describe how “out” they are about having sex with men [53] and being a transgender woman, respectively. Participants responded on a scale of 1 to 7 (1=not out to anyone, 4=out to about half the people I know, and 7=out to everyone). History of discrimination related to same-sex sexual behavior was assessed among MSM participants only by asking “In your day to day life, how often does discrimination related to your sexual orientation/having sex with men happen to you?” (0=never, 1=less than once a year, 2=a few times a year, 3=a few times a month, 4=at least once a week, and 5=almost every day).

#### Outcome of Interest

Web-based sex seeking was measured by asking, “In the past 4 months, how many different men did you meet online with the intention of having sex?” Participants who reported meeting at least one man online with the intention of having sex were classified as Web-based sex seekers.

#### Other Covariates

Sociodemographic data were collected on participants’ gender identity (0=transgender female and 1=male), age (in years), highest level of education completed (1=cannot read or write, 2=some grade school but no certificate, 3=grade school, 4=some secondary school but no certificate, 5=secondary school, 6=some high school but no certificate, 7=high school, 8=some university but no title, 9=university, and 10=advanced degree such as doctorate or masters), average monthly income in Mxn pesos in the past 4 months (1=no income, 2<Mxn $1000, 3=Mxn $1000-Mxn $1499, 4=Mxn $1500-Mxn $1999, 5=Mxn $2000-Mxn $2499, 6=Mxn $2500-Mxn $2999, 7=Mxn $3000-Mxn $3500, and 8>Mxn $3500, which was dichotomized at Mxn $3000 based on the national monthly well-being lines representing federal poverty limits for urban areas during our study period) [54], years of residence in Tijuana, and marital status (1=married to a woman, 2=married to a man, 3=separated or filing for divorce, 4=divorced but not remarried, 5=widowed but not remarried, 6=never married, 7=common-law marriage with female partner, and 8=common-law marriage with male partner). Social support was measured via 8 items that make up the Modified Medical Outcome Study Social Support Survey assessing help and support received from others (eg, “If you needed it, how often is someone available to turn to for suggestions about how to deal with a personal problem?” and “If you needed it, how often is someone available to help with daily chores if you were sick?”; Cronbach alpha=.97) [55]. Participants provided their level of agreement using 5-point Likert scale responses (1=none of the time, 2=a little of the time, 3=some of the time, 4=most of the time, and 5=all of the time). A mean score was calculated from item responses and transformed to a 100-point scale, where higher values indicate greater social support [56]. Participants were asked with how many people they engaged in sexual (vaginal or anal) intercourse in the past 4 months and whether they had given or received money, drugs, or other goods in exchange for sex in the past 4 months (0=did not give or receive money, drugs, or other goods for sex and 1=gave or received money, drugs, or others goods for sex), which has been shown to be a reliable period of recall for self-reported sexual contact and behavior data [57-59]. HIV testing in the past 12 months was assessed by asking 2 questions: “Have you ever been tested for HIV?” (0=no and 1=yes) and “How long ago was your last HIV test?” Participants were categorized as undergoing HIV testing in the past 12 months if they responded that they had ever been tested for HIV and their last HIV test was within 12 months of the interview date.

### Statistical Analysis

This analysis includes all *Proyecto Redes* participants (n=396; excluding 11 identified via RDS or VBS more than once who later tested HIV positive and enrolled in *Proyecto Enlaces*) and newly diagnosed HIV-positive participants enrolled in *Proyecto Enlaces* (n=165). Analyses considering 3 of the 5 stigma measures (ie, traditional *machismo*, history of discrimination related to same-sex sexual behavior, and sexual orientation) were restricted to specific subgroups of our sample because (1) HIV-positive *Proyecto Enlaces* participants completed baseline and supplemental (approximately 2 weeks post baseline when they returned for their confirmatory HIV test results) surveys, whereas HIV-negative *Proyecto Redes* participants only completed baseline surveys and (2) some survey questions were phrased for both MSM and TW, whereas others were only phrased for MSM. Data on traditional *machismo* and history of discrimination related to same-sex sexual behavior were collected via the supplemental survey among HIV-positive *Proyecto Enlaces* participants. As 27.3% (45/165) of *Proyecto Enlaces* participants did not return to complete the supplemental survey, analyses examining traditional *machismo* and history of discrimination related to same-sex sexual behavior were restricted to HIV-negative *Proyecto Redes* participants to reduce the potential for selection bias that could have been introduced by only including those *Proyecto Enlaces* participants who had returned. Analyses examining history of discrimination were then further restricted to MSM participants because the phrasing of the question was specific to experiences of MSM and not TW (Multimedia Appendix 1). Finally, analyses examining sexual orientation were restricted to MSM because we hypothesized that MSM and TW less affected by stigma would identify as gay or homosexual and heterosexual, respectively. Restricting these analyses to MSM allowed us to examine sexual orientation without potentially including participants who were more or less affected by stigma in the same category.

Descriptive statistics were calculated to characterize the study population by Web-based sex seeking. Next, bivariate associations between factors that shape or are influenced by stigma toward sexual and gender minorities were examined using linear and logistic regression models for continuous and categorical dependent variables, respectively. Separate unadjusted and adjusted logistic regression models were then used to examine the total effect of each stigma measure on Web-based sex seeking. A review of the literature was conducted to identify covariates that may confound the total effects of interest. Directed acyclic graphs (DAGs) [60] were then generated to depict interrelationships among each exposure of interest, Web-based sex seeking, and the identified covariates. Westreich et al have shown that it is inappropriate to interpret effect estimates for exposures of interest from a single adjusted model (ie, one that includes all exposures of interest) as their total effects on the outcome of interest because adjusting for an exposure of interest that lies on the causal pathway between another exposure of interest and the outcome of interest would yield an estimate of the direct effect of that exposure of interest on the outcome of interest [61]. Therefore, given the highly interrelated nature of our exposures of interest and the cross-sectional nature of our data (making it difficult to determine the directionality of the relationships among our exposures of interest), our DAGs considered 1 exposure of interest at a time to facilitate the identification of confounders for inclusion in adjusted models for the total effect of each exposure of interest on Web-based sex seeking. On the basis of these DAGs, sociodemographics (gender identity, age, education, and monthly income) and social support were identified as confounders and selected for inclusion in adjusted models. We were unable to stratify models by gender identity because of the small number of TW included in our sample (n=32). However, as controlling for gender identity in our adjusted models could have resulted in sparse data bias (because of the small number of TW), we conducted a sensitivity analysis excluding TW and found qualitatively similar results. In another sensitivity analysis, we examined the potential impact of grouping MSM who reported being unsure of their sexual orientation with those who did not identify as gay by excluding those who reported being unsure of their sexual orientation from models examining the effect of sexual orientation on Web-based sex seeking, and we found qualitatively similar results. Finally, we examined whether any of the relationships of interest were modified by HIV status or age by including product terms between the potential effect measure modifier and the exposures of interest in each of their respective models. Unstratified results are presented because none of the product terms were statistically significant. All statistical analyses were conducted using SAS V.9.3. (SAS Institute, Inc).

## Results

### Sample Characteristics

Approximately half of our sample was recruited via VBS (311/561, 55.4%; Table 1). Most participants identified as male (529/561, 94.3%), and participants had a mean age of 37 years (SD 11.2). Nearly half of the participants (239/561, 42.6%) reported at least a high school education, and 69.2% (387/561) of the participants reported an average monthly income of at least Mxn $3000 (approximately US $150). A total of 29.4% (165/561) of participants reported seeking sex partners on the Web in the past 4 months.

**Table 1:** Characteristics of men who have sex with men and transgender women in Tijuana, Mexico, by Web-based sex seeking in the past 4 months (N=561).

Characteristics^a^				Total (N=561)	Web-based sex seeking (n=165)	No Web-based sex seeking (n=396)	*P* value^b^
**Stigma measures**							
	Traditional *Machismo* (range: 1-4)^c^, mean score (SD)			2.1 (0.4)	1.9 (0.4)	2.2 (0.4)	<.001
	Internalized stigma related to same-sex sexual behavior or gender identity (range: 9-45), mean score (SD)			23.9 (8.7)	20.5 (8.4)	25.3 (8.3)	<.001
	**Sexual orientation^d^, n (%)**						<.001
		Gay or homosexual		202 (38.2)	96 (61.9)	106 (28.3)	
		Bisexual, heterosexual, or not sure		327 (61.8)	59 (38.1)	268 (71.7)	
	Outness about same-sex sexual behavior or gender identity (range: 1-7), mean score (SD)			4.2 (2.5)	5.1 (2.1)	3.8 (2.5)	<.001
	History of discrimination related to same-sex sexual behavior^e^, n (%)			147 (38.4)	52 (46.0)	95 (35.2)	.05
**Covariates**							
	**Sociodemographics**						
		**HIV status, n (%)**					.61
			Newly diagnosed HIV positive (*Proyecto Enlaces*)	165 (29.4)	46 (27.9)	119 (30.1)	
			HIV negative (*Proyecto Redes*)	396 (70.6)	119 (72.1)	277 (70.0)	
		Cisgender male, n (%)		529 (94.3)	155 (93.9)	374 (94.4)	.81
		Age (years), mean (SD)		37.0 (11.2)	31.0 (9.0)	39.6 (11.0)	<.001
		Completed at least a high school education, n (%)		239 (42.6)	96 (58.2)	143 (36.1)	<.001
		Average monthly income ≥Mxn $3000, n (%)		387 (69.2)	135 (82.3)	252 (63.8)	<.001
		Years of residence in Tijuana, mean (SD)		12.7 (12.6)	14.9 (11.3)	11.7 (13.0)	.004
		**Marital status, n (%)**					.01
			Married, including common-law marriage^f^	83 (14.8)	21 (12.7)	62 (15.7)	
			Separated or divorced	72 (12.9)	12 (7.3)	60 (15.2)	
			Widowed	6 (1.1)	0 (0.0)	6 (1.5)	
			Never married	399 (71.3)	132 (80.0)	267 (67.6)	
	Social support (range: 0-100), mean score (SD)			56.6 (34.6)	67.1 (27.6)	52.3 (36.3)	<.001
	Sex partners (past 4 months), mean (SD)			11.9 (32.7)	11.3 (29.0)	12.1 (34.2)	.76
	Exchanged money, drugs, or other goods for sex (past 4 months), n (%)			271 (48.7)	51 (30.9)	220 (56.1)	<.001
	Tested for HIV (past 12 months), n (%)			224 (43.4)	76 (50.0)	148 (40.7)	.05
	**Recruitment method, n (%)**						.30
		Respondent-driven sampling		250 (44.6)	68 (41.2)	182 (46.0)	
		Venue-based sampling		311 (55.4)	97 (58.8)	214 (54.0)	

^a^Numbers may not sum to total because of missing data; percentages may not sum to 100 because of rounding.

^b^*P* value from chi-square test (if categorical) or 2-sided *t* tests (if continuous).

^c^Restricted to HIV-negative participants (n=396).

^d^Restricted to men who have sex with men participants (n=529).

^e^Restricted to HIV-negative men who have sex with men participants (n=383).

^f^A total of 43 men who have sex with men reported being married to a woman, 34 men who have sex with men reported being married to a man, and 6 transgender women reported being married to a man.

### Bivariate Associations Between Stigma Measures

Bivariate associations between factors that shape or are influenced by stigma toward sexual and gender minorities were in the expected directions (Table 2). Positive associations were observed between traditional *machismo* and internalized stigma related to same-sex sexual behavior or gender identity (beta coefficien*t* =.019; 95% CI 0.015 to 0.024). Outness about same-sex sexual behavior or gender identity was positively associated with gay identification (beta coefficient=2.35; 95% CI 1.96 to 2.73) and history of discrimination related to same-sex sexual behavior (beta coefficien*t* =.93; 95% CI 0.42 to 1.43). Traditional *machismo* was negatively associated with outness about same-sex sexual behavior or gender identity (beta coefficient=−.04; 95% CI −0.06 to −0.03) and gay identification (beta coefficient=−.25; 95% CI −0.33 to −0.17). Internalized stigma related to same-sex sexual behavior or gender identity was inversely associated with outness about same-sex sexual behavior or gender identity (beta coefficient=−1.84; 95% CI −2.08 to −1.59) and sexual orientation (beta coefficient=−8.94; 95% CI −10.25 to −7.63).

**Table 2:** Associations between factors that shape or are influenced by stigma among men who have sex with men and transgender women in Tijuana, Mexico.

Stigma Measures	Traditional *machismo*^a^		Internalized stigma^a^		Outness^a^			Gay identifying^b^	
	β^c^	95% CI	β	95% CI	β	95% CI	β		95% CI
Internalized stigma	.019^d^	0.015 to 0.024	—^e^	—	—	—	—		—
Outness	−.04^d^	−0.06 to −0.03	−1.84	−2.08 to −1.59	—	—	—		—
Gay identifying	−.25^f^	−0.33 to −0.17	−8.94^g^	−10.25 to −7.63	2.35^g^	1.96 to 2.73	—		—
History of discrimination	−.01^f^	−0.10 to 0.07	.29^f^	−1.48 to 2.07	.93^f^	.42 to 1.43	.41^f^		−0.03 to 0.85

^a^Unadjusted linear regression.

^b^Unadjusted logistic regression.

^c^Beta coefficient.

^d^Restricted to HIV-negative participants.

^e^Not Applicable.

^f^Restricted to HIV-negative men who have sex with men participants.

^g^Restricted to men who have sex with men participants.

### Stigma Measures and Web-Based Sex Seeking

On average, Web-based sex seekers reported less endorsement of traditional *machismo* values (mean 1.9 vs mean 2.2, range 1 to 4; *P*<.001; Table 1), less internalized stigma related to same-sex sexual behavior or gender identity (mean 20.5 vs mean 25.3, range 9 to 45; *P*<.001), and they were more out about their same-sex sexual behavior or gender identity (mean 5.1 vs mean 3.8, range 1 to 7; *P*<.001). Compared with MSM participants who did not seek sex partners on the Web, a greater proportion of Web-based sex seekers identified as gay (61.9% vs 28.3%, *P*<.001) and reported a history of discrimination related to their same-sex sexual behavior (46.0% vs 35.2%; *P*=.05).

After adjusting for sociodemographics and social support (Table 3), the odds of Web-based sex seeking were lower for participants who reported greater endorsement of traditional *machismo* values (adjusted odds ratio [AOR] 0.36, 95% CI 0.19 to 0.69) and greater levels of internalized stigma related to their same-sex sexual behavior or gender identity (AOR 0.96, 95% CI 0.94 to 0.99), whereas the odds of Web-based sex seeking were higher for those who reported greater outness about their same-sex sexual behavior or gender identity (AOR 1.17, 95% CI 1.06 to 1.28). Among MSM participants, the odds of Web-based sex seeking were higher for gay-identifying participants (AOR 2.13, 95% CI 1.36 to 3.33) and those who reported a history of discrimination related to their same-sex sexual behavior (AOR 1.83, 95% CI 1.08 to 3.08).

**Table 3:** Associations among factors that shape or are influenced by stigma and Web-based sex seeking among men who have sex with men and transgender women in Tijuana, Mexico.

Stigma measures	Unadjusted odds ratio (95% CI)	Adjusted^a^ odds ratio (95% CI)
Traditional *machismo*^b^	0.20 (0.11 to 0.36)	0.36 (0.19 to 0.69)
Internalized stigma related to same-sex sexual behavior or gender identity	0.93 (0.91 to 0.96)	0.96 (0.94 to 0.99)
Gay identifying^c^	4.11 (2.77 to 6.10)	2.13 (1.36 to 3.33)
Outness about same-sex sexual behavior or gender identity	1.25 (1.15 to 1.35)	1.17 (1.06 to 1.28)
History of discrimination related to same-sex sexual behavior^d^	1.57 (1.01 to 2.45)	1.83 (1.08 to 3.08)

^a^Adjusted models for each stigma measure of interest included the following: gender identity (male vs transgender female in models not restricted to men who have sex with men), age (years), education (less than a high school education vs at least a high school education), monthly income (<Mxn $3000 vs ≥Mxn $3000 ), years of residence in Tijuana (years), and social support (score on the Modified Medical Outcome Study Social Support Survey) [55].

^b^Restricted to HIV-negative participants.

^c^Restricted to men who have sex with men participants.

^d^Restricted to HIV-negative men who have sex with men participants.

## Discussion

We examined the relationship between stigma and Web-based sex seeking using cross-sectional data collected from MSM and TW in Tijuana, Mexico. Nearly one-third of our sample reported seeking sex partners on the Web in the past 4 months, which is consistent with estimates from Peru (36%) [11] but is not as high as those from the United States (44%) [12], Africa (39%-62%) [13,14], or Asia (41%-77%) [15-18].

Our a priori hypothesis was that Web-based sex seeking would be more common among MSM and TW most affected by stigma toward sexual and gender minorities. However, for the most part, our findings do not support this hypothesis. More specifically, participants who met sex partners on the Web reported less agreement with traditional *machismo* values, less internalized stigma related to same-sex sexual behavior or gender identity, were more gay identifying, and were more out about their same-sex sexual behavior or gender identity compared with those who did not seek sex on the Web. Prior research suggests that MSM and TW most affected by stigma often isolate themselves from other sexual and gender minorities to conceal their minority status [28,32]. As such, those most affected by stigma in our sample may be less connected to communities of MSM and TW and, thus, less aware of or less comfortable using sex-seeking websites and apps used by MSM and TW. Although MSM participants with a history of discrimination were more likely to seek sex partners on the Web, as was our a priori hypothesis, it seems plausible, given our other findings, that this result may have an alternative explanation. As reported in other settings [13,14], we initially hypothesized that MSM with a history of discrimination would be more likely to seek sex partners on the Web, partly to avoid future discrimination that they could face by meeting sex partners in traditional physical venues. However, given our other findings, it is possible that having a history of discrimination was associated with Web-based sex seeking in our sample because MSM who identify as gay or are more out about their same-sex sexual behavior (both of which were positively associated with Web-based sex seeking and history of discrimination) may be more susceptible to discrimination.

Overall, our findings suggest that Web-based sex seeking is more common among MSM and TW in Tijuana who are less affected by stigma toward sexual and gender minorities. Our findings are similar to those from exploratory studies in Peru [11], China [15,17,18], Lesotho [13], and Swaziland [13], which found that MSM who used the Web to meet sex partners were more gay identifying [11,13,17,18]. Our findings also align with those from a study in Nigeria [14], which found that MSM who sought sex partners on the Web were more likely to report a history of discrimination. We also expanded on previous literature by examining a wider range of factors that shape or are influenced by stigma, including a social norm that influences societal stigma (traditional *machismo*) and internalized stigma related to same-sex sexual behavior or gender identity. Including a broader scope of factors that shape or are influenced by stigma provides a more multidimensional understanding of the role of stigma with respect to Web-based sex seeking, which may have been underappreciated by prior studies that considered only 1 or 2 factors [24,25].

Our study has several limitations. First, because of the cross-sectional design of our study, we cannot establish a temporal relationship between the exposures of interest and Web-based sex seeking. Therefore, we cannot infer that the observed associations represent causal associations. Second, our use of nonprobability sampling methods may limit the generalizability of our findings. These sampling methods, however, were critical to our ability to recruit MSM and TW who are socially marginalized and often hidden in Tijuana. However, even with the use of these sampling methods, TW were underrepresented in our sample, which precluded us from stratifying our analyses by gender identity. As such, our results do not shed light on potential differences in Web-based sex seeking or its relationship with stigma between MSM and TW, which should be examined in future research. Third, our assessment of Web-based sex seeking was exclusive to meeting male partners. As a result, we may have underestimated the prevalence of Web-based sex seeking among MSM and TW in Tijuana because those who only met TW partners on the Web would not have been classified as Web-based sex seekers. Fourth, we did not collect data on smartphone or computer use, both of which are likely associated with Web-based sex seeking and should be considered in future research. Fifth, although surveys were interviewer administered to alleviate respondent burden, interviewer-administered surveys may have introduced social desirability bias if participants underreported sensitive information related to the exposures of interest or Web-based sex seeking. However, to minimize the potential for social desirability bias, all interviewers had extensive experience working with sexual and gender minorities in Tijuana and were trained to create a nonjudgmental environment and build rapport with participants to enhance their comfort with respect to responding openly and honestly. Finally, it is possible that poor recall could have led to the misclassification of history of discrimination related to same-sex sexual behavior and Web-based sex seeking in the past 4 months. However, such misclassification was likely nondifferential with regard to the outcome or exposure and, thus, may have only biased effect estimates toward the null.

Despite these limitations, our findings suggest that Web-based sex seeking is relatively common among MSM and TW in Tijuana and that it may be feasible to leverage Web-based sex-seeking platforms to engage these vulnerable populations in HIV prevention and treatment services. However, such Web-based interventions may still poorly engage those most affected by stigma toward sexual and gender minorities, given that within our sample they were least likely to seek sex on the Web. Further research is needed to identify effective and acceptable strategies to link MSM and TW most affected by stigma to HIV prevention and treatment services. As trends of Web-based sex seeking among nationally representative samples of MSM in the United States have dramatically increased over time [12], future research should also monitor changes in the use of Web-based sex-seeking platforms among MSM and TW in LMIC.

## Appendix

Multimedia Appendix 1 Scale items and survey questions for the 5 stigma measures used in this analysis.

## Abbreviations

AOR: adjusted odds ratio
DAGs: directed acyclic graphs
LMIC: low- and middle-income countries
MSM: men who have sex with men
MXN: Mexican pesos
RDS: respondent-driven sampling
TW: transgender women
VBS: venue-based sampling
