# Supplementary material for: Stigma and Web-Based Sex Seeking Among Men Who Have Sex With Men and Transgender Women in Tijuana, Mexico: Cross-Sectional Study
Source: JMIR Public Health Surveill. 2020 Jan 30;6(1):e14803. doi: 10.2196/14803 (PMC7055757; doi:10.2196/14803)
Supplement: Multimedia Appendix 1 [file publichealth_v6i1e14803_app1.docx]

| Multimedia Appendix 1. Scale items and survey questions for stigma measures | |
| --- | --- |
| 10-item Traditional *Machismo* scale | |
| How much do you agree with the following statement… | |
|  | Men are superior to women. |
|  | In a family, a father's wish is law. |
|  | The birth of a male child is more important than a female child. |
|  | It is important for men not to be the weakest in a group. |
|  | Real men never let down their guard. |
|  | It would be shameful for a man to cry in front of his children. |
|  | A man should be in control of his wife. |
|  | It is necessary for a man to fight when challenged. |
|  | It is important for women to be beautiful. |
|  | The bills (electric, phone, etc.) should be in the man's name. |
| Response options (choose one): strongly disagree, disagree, agree, strongly agree | |
| 9-item internalized stigma scale related to same-sex sexual behavior (MSM only) | |
| How much do you agree or disagree with… | |
|  | I have tried to stop being attracted to men in general. |
|  | If someone offered me the chance to stop having sex with men, I would accept the chance. |
|  | I wish I didn't have sex with men. |
|  | I feel that having sex with men is a personal shortcoming for me. |
|  | I would like to get professional help in order to change my sexual attraction from men to women exclusively. |
|  | I have tried to become more sexually attracted to women. |
|  | I often feel that it is best to avoid personal or social involvement with men who have sex with men. |
|  | I feel alienated from myself because I have sex with men. |
|  | I wish that I could develop more erotic feelings about women. |
| Response options (choose one): strongly disagree, disagree, neither agree nor disagree, agree, strongly agree | |
| Adapted 9-item internalized stigma scale related to gender identity (TW only) | |
| How much do you agree or disagree with… | |
|  | I have tried to stop identifying as a woman in general. |
|  | If someone offered me the chance to identify as a man, I would accept the chance. |
|  | I wish I weren't a transgender woman. |
|  | I feel that being a transgender woman is a personal shortcoming for me. |
|  | I would like to get professional help in order to change my gender identity from female to male exclusively. |
|  | I have tried to become more sexually attracted to women. |
|  | I often feel that it is best to avoid personal or social involvement with other transgender women. |
|  | I feel alienated from myself because I am a transgender woman. |
|  | I wish that I could develop more erotic feelings about women. |
| Response options (choose one): strongly disagree, disagree, neither agree nor disagree, agree, strongly agree | |
| Single item measuring sexual orientation (MSM only) | |
|  | What is your sexual orientation? |
| Response options (choose one): gay/homosexual, heterosexual, bisexual, not sure | |
| Single item measuring outness about same-sex sexual behavior (MSM only) | |
|  | Please choose the number that best describes how "out" you currently are about having sex with men. By "out", we mean you let others know that you are sexually attracted to men. So, on a scale of 1 to 7, with 1 meaning that you are "not out to anyone", 4 meaning that you are "out to half the people you know” and 7 meaning "out to everyone", how "out" are you? |
| Response options on a continuous scale from 1 to 7 | |
| Adapted single item measuring outness about gender identity (TW only) | |
|  | Please choose the number that best describes how "out" you currently are about being a transgender woman. By "out", we mean you let others know that you identify as female. So, on a scale of 1 to 7, with 1 meaning that you are "not out to anyone", 4 meaning that you are "out to half the people you know” and 7 meaning "out to everyone", how "out" are you? |
| Response options on a continuous scale from 1 to 7 | |
| Single item measuring history of discrimination related to same-sex sexual behavior (MSM only) | |
|  | In your day-to-day life, how often does this type of discrimination happen to you? Discrimination related to your sexual orientation/having sex with men |
| Response options (choose one): never, less than once a year, a few times a year, a few times a month, at least once a week, almost every day | |
